# Missing school during period: perspectives of adolescent schoolgirls in Cross River State, Nigeria

**DOI:** 10.11604/pamj.2022.42.65.28244

**Published:** 2022-05-24

**Authors:** Olaide Bamidele Edet, Philip Etabee Macdonald Bassey, Ekpoanwan Esienumoh Esienumoh, Antor Odu Ndep

**Affiliations:** 1 Department of Nursing Science, College of Medical Sciences, University of Calabar, Calabar, Cross River State, Nigeria,; 2 Department of Public Health, College of Medical Sciences, University of Calabar, Calabar, Cross River State, Nigeria

**Keywords:** Adolescents, menstruation, school-absenteeism, schoolgirls, menstrual hygiene management

## Abstract

**Introduction:**

adolescent in-schoolgirls often experience stigmatization, physical and psychological stress during their menstrual period that causes them to miss school. Menstruation-induced school absenteeism is preventable. The purpose of the study was to assess the factors associated with school absenteeism by both urban and rural school-going adolescent girls during their period.

**Methods:**

adolescent female students in Junior and Senior Secondary classes, who have attained menarche, participated in the study. A researcher designed, pretested and validated self-administered questionnaire, consisting of questions related to perception about menstruation and reasons for missing school during menstruation was used for data collection. Data were analyzed using SPSS software. Descriptive data were presented using percentages, while the association between the variables of interest, were verified using Chi-Square test.

**Results:**

mean age of the girls was 14.4 (±1.8) years while mean age at menarche was 12.2 (±1.3) years. Although respondents identified several factors as responsible for school absenteeism, however, only fear of ridicule by other students (p ≤ 0.001) and unavailability of sanitary towels (p = 0.006) were significantly associated with missing school. Lack of sanitary towels was significantly associated with location (p = 0.012) and socio-economic status of mothers (0.006); while perception of menstruation as a disease was associated with feeling of tiredness and discomfort (p = 0.017).

**Conclusion:**

findings have shown that school absenteeism during menstruation is a serious problem among respondents capable of adversely affecting their academic performance. Access to sanitary towels and WASH facilities should be provided in schools to create an enabling environment to motivate school attendance by the adolescent girls.

## Introduction

Adolescents by the World Health Organization (WHO) definition are young people in the age group between 10 and 19 years [1]. This period is a transition phase for females, and is characterized by rapid physical growth, physiological transformations as well as psychological and behavioral changes [1, 2]. Menarche the first menstrual period of a girl is one of the most important physiological changes in the life of the female and marks the beginning of the reproductive life of the female [3]. It usually occurs between the ages of 11 and 14 years, with a mean onset around 13 years, which varies between developed and developing settings [4]. Menstruation in some cultures is shrouded in taboos, myths, misconceptions and prohibitions based on the notion that the menstrual discharge is unclean or dirty [5]; thereby putting an undue psychological stress on the female in these societies [6]. Within the school setting, adolescent school girls are confronted by the same negative societal gender stereotypes and wrong perceptions about menstruation including physical, environmental and economic barriers to decent menstrual hygiene management [7], which collectively put the girl child under pressure and stress when they have their monthly period, causing them to temporarily absent themselves from school. From available search, no published study was found emanating from Cross River State, Nigeria, determining how menstruation affects school attendance by adolescent in-schoolgirls. The purpose of the study is assessing the misconceptions, knowledge, institutional or systemic gaps about menstruation and menstrual hygiene management that impede school attendance during menstrual periods with a view to providing appropriate health education intervention to address identified gaps.

This study was undertaken to identify self-reported factors associated with missing school on account of menstruation among adolescent in-schoolgirls in Cross River State. 1) To determine the factors (misconceptions, knowledge, institutional or systemic gaps about menstruation and menstrual hygiene management) that impede school attendance during menstrual periods with a view to providing appropriate health education intervention to address identified gaps. 2) To examine the influence of the socio-economic status of the mothers in relation to the adolescent in-schoolgirls on access to sanitary wares. To guide the study, the research questions and hypotheses listed below were formulated.

The research questions were: 1) What is the proportion of respondents who provided the underlisted reasons for school absenteeism during menstruation by location in Cross River State? a) Fear of staining their school uniform; b) Fear of being ridiculed by other students; c) Lack of sanitary towels; d) Place for girls to wash and change their sanitary towels; e) Disposal of menstrual materials; f) Menstrual pains. 2) In what way does the socio-economic status of the mothers of the in-school adolescent girls in Cross River State influence respondents´ access to sanitary wares?

The hypotheses were: 1) there is no statistically significant difference between menstruating rural and urban adolescent in-schoolgirls in Cross River State who miss school due to the ridicule of other students. 2) There is no statistically significant difference between rural and urban-based adolescent in-schoolgirls in Cross River State missing school due to the fear of staining their school uniform. 3) There is no statistically significant difference between rural and urban-based adolescent in-schoolgirls in Cross River State missing school due to lack of sanitary towels. 4) There is no statistically significant difference between rural and urban-based adolescent in-schoolgirls in Cross River State missing school due to menstrual pain. 5) There is no statistically significant association of the lack of sanitary towels and the socio-economic status of the mothers of in-school adolescents in both rural and urban settings of Cross River State who miss school during their periods.

## Methods

**Study design:** an analytic cross-sectional study using a self-administered questionnaire was conducted among randomly selected adolescent in-schoolgirls. The study was conducted from February 2^nd^ 2016 to March, 2016.

**Study setting:** the study setting comprised 7 high schools that included 4 public and 3 private schools drawn from one urban and one rural Local Government Area of Cross River State.

**Study population:** the study population included 1006 adolescent in-school female students, age 10-18 years selected from both Junior and Senior Secondary classes of government and privately owned secondary schools located in both rural and urban local government areas (LGAs) of Cross River State, Nigeria.

**Instrument for data collection:** researcher designed structured self-administered questionnaire was used for data collection. The questionnaire consisted of questions related to the girls´ perceptions about menstruation and menstrual hygiene, and their reasons for missing school during menstruation. The instrument was pretested among students from equivalent schools in non-participating LGAs, using a test-retest approach to establish the reliability of the instrument. A Cronbach´s Alpha (Coefficient Alpha) of 0.704 obtained was considered acceptable for the study [8]. Findings from the pre-test were used to revise the questionnaire to make it more comprehensible.

**Figure 1:**
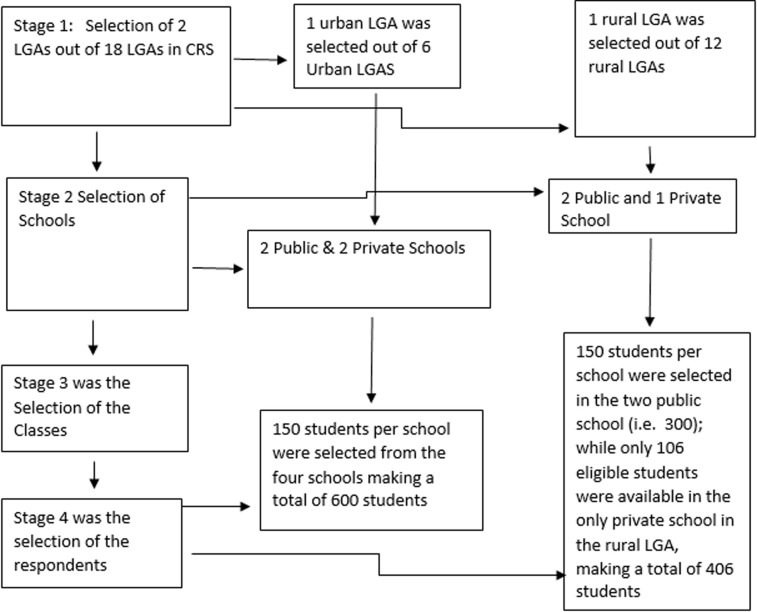
flow chart showing the multi-stage phases in selecting the respondents

**Sampling method:** a multi-stage sampling technique was applied in selecting the respondents. In the first stage, one urban and one rural LGA were randomly selected from a line list of six (6) urban and twelve (12) rural LGAs respectively. For the second stage, two public schools were randomly selected from the urban and rural LGAs respectively, while two private schools were selected from the urban LGA along with the only available private school in the rural LGA. In all, a total of 7 schools were selected as shown in the Flow Chart (Figure 1). A simple random sampling method using balloting was adopted in selecting the students from both the junior and senior classes.

**Research variables:** the explanatory /independent variables are the self-perceived reasons by respondents for being absent from school which include: fear of staining their school uniform, fear of being ridiculed by other students, lack of sanitary towels, or unavailability of a place for girls to wash and change their sanitary towels or dispose their menstrual materials, and the experience of menstrual pains. The dependent/outcome variable is missing school/school absenteeism during menses. The study is a self-report by an in-school adolescent girl on the reason for staying away from school during the school session for a minimum of 1 day or more as a result of the menstrual experience.

**Sample size determination:** this was determined by using Cohen´s [9] formula. As the baseline of an intervention study, this formula is considered to be appropriate for selecting the required study sample based on the following assumptions: the use of two-tailed (non-directional) hypotheses: Adoption of a critical (Za/2) value of 1.96 for (0.05) level of significance and a (95% confidence interval); The power (ability of test to detect type 11 error) which was set at 80%; A margin of error of 15% (i.e. difference between proportions that will be considered relevant or significant) if it exists, was set; A prevalence (P) of menstrual hygiene practice (0.40) from previous study [10] was used while Cohen´s formula) [9] was used for the calculation to arrive at 141 participants per school. The calculated sample size per school was increased to 150 participants to make up for non-response.

**Data collection and analysis:** prior to the administration of the questionnaire, the purpose of the study was first explained to the girls and confidentiality was also assured, the procedure for the completion of the questionnaire was equally explained to them. The self-administered questionnaires were completed within 40 minutes and were retrieved after the allotted time. The objective of the data analysis was to provide answers to the research questions through the testing of the hypotheses. Data entry and analysis was done using SPSS software version 22 (IBM Corporation, New York, USA). Both descriptive and inferential statistics were used in reporting the results of the study. Frequency data were expressed as percentages while, Chi-Square test was used to assess the association between categorical study variables. Hypotheses were tested at 0.05 level of significance. Results are presented using tables and figures.

### Ethical considerations

Ethical approval for the study was given by the Cross River State Ethical Review Board of the Ministry of Health with reference number CRS/MH/CGS/E-H/018/Vol. II/122). The authorities of the selected schools gave administrative permission for the study while the parents of the students gave their written consent.

## Results

All the 1006 questionnaire administered to the students were retrieved; however only 673 respondents provided valid responses to the questions on why they missed school during their menstrual period.

**Demographic characteristics of the respondents:** most of 600 (59.6%) of the respondents were urban-based, while 406 (40.4%) were rural-based. A detailed demographic characteristic of the respondents has been published earlier [11].

### Factors responsible for school absenteeism during menstruation by the respondents

Table 1 provides the main factors reported by the adolescent schoolgirls´ as determinants of absenteeism from school during the menstrual period. These include fear of being ridiculed, lack of sanitary towels, fear of staining the school uniform, lack of bins to dispose of sanitary towels, lack of gender-sensitive toilets for girls to wash and change, feelings of tiredness and discomfort and menstrual pains. Overall, out of the 1006 respondents, a varied number of students responded to the different reasons why girls miss school. It should be borne in mind that multiple responses were allowed. Fear of staining school uniform attracted the highest response of 673 corresponding to 67% of the respondents. Lack of sanitary towels attracted only 612 responses, constituting the least response. Five hundred and fifty-two (54.9%) of the respondents indicated that the fear of being ridiculed contributed to their not attending school during their period. Five hundred and fourteen (51.1%) of the girls agreed that lack of sanitary towels accounted for school absenteeism during their menstrual period. Five-hundred and twenty-two (51.9%) of the girls acknowledged that the fear of staining their school uniform compelled them to stay at home, while 480 (47.7%) and 475 (47.2%) respectively, reported that they stayed away from school during their period due to the non-availability of a place for girls to wash and change as well as the lack of a place to dispose of their soiled sanitary materials (Table 1).

**Table 1:** proportion of respondents who provided various reasons for school absenteeism during menstruation (n=1006)

Reasons for missing school	Response to the determinants of school absenteeism during menses as a proportion of total 1006 respondents			Disaggregated responses for urban-based respondents (n=600)		Disaggregated responses for rural-based respondents (n=406)	
	Agree	Disagree	Total	Agree	Disagree	Agree	Disagree
Fear of being ridiculed	552 (54.9%)	78 (7.8%)	630 (62.7%)	379 (63.2%)	36 (6.0%)	173 (42.6%)	42 (10.3%)
Fear of staining school uniform	522 (51.9%)	151 (15.0%)	673 (66.9%)	346 (58.0%)	88 (14.7%)	176 (43.3%)	63 (15.5%)
Lack of sanitary towels	514 (51.1%)	98 (9.7%)	612 (60.8%)	356 (59.3%)	54 (9.0%)	158 (38.9%)	44 (10.8%)
No place for girls to wash and change	480 (47.7%)	163(16.2%)	643 (63.9%)	324 (54.0%)	101 (16.8%)	156 (38.4%)	62 (15.3%)
No place for girls to dispose sanitary material	475 (47.2%)	156 (15.5%)	631 (62.7%)	316 (52.7%)	110 (18.3%)	159 (39.2%)	46 (11.3%)
Menstrual pain	449 (44.6%)	220 (21.9%)	669 (66.5%)	304 (50.7%)	133 (22.2%)	145 (35.7%)	87 (21.4%)

### Reasons for missing school during menstrual period by location of respondents

This study involved respondents selected from both urban and rural settings, who provided various perceived reasons for school absenteeism during their menstrual period. As shown in Table 2, 371 (60.1%) urban and 173 (27.5%) rural adolescent in-schoolgirls, respectively, agreed that being ridiculed by fellow students was a good enough reason for missing school during their period. 346 (51.4%) urban and 176 (26.1%) rural-based-students were also in agreement that the fear of staining their school school uniforms accounted for their staying off school during their period. With regard to their having access to sanitary towels, 356 (58.2%) urban and 158 (25.2%) rural respondents respectively indicated that the lack of sanitary towels was responsible for their absence from school. The non-availability of a place for the disposal of their menstrual pads was also an important factor that contributed to school absenteeism, 312 (50.1%) of the urban girls reported that not having a place in school where they can conveniently dispose of their soiled sanitary wares was a problem, while only 159 (25.2%) of the rural-based students saw it as a major issue (Table 2).

**Table 2:** actual respondents who provided reasons for school absenteeism during menstruation by location

Reasons for missing School	Overall response			Urban respondents		Rural respondents	
	Agree	Disagree	Total	Agree	Disagree	Agree	Disagree
Fear of being ridiculed	552 (87.6%)	78 (12.4%)	630 (100%)	379 (60.1%)	36 (5.7%)	173 (27.5%)	42 (6.7%)
Fear of staining school uniform	522 (77.6%)	151 (22.4%)	673 (100%)	346 (51.4%)	88 (13.1%)	176 (26.1%)	63 (9.4%)
Lack of sanitary towels	514 (84.0%)	98 (16.0%)	612 (100%)	356 (58.2%)	54 (8.8%)	158 (25.8%)	44 (7.2%)
No place for girls to dispose sanitary materials	475 (75.3%)	156 (24.7%)	631 (100%)	316 (50.1%)	110 (17.4%)	159 (25.2%)	46 (7.3%)
Menstrual pain	449 (67.1%)	220 (32.9%)	669 (100%)	304 (45.4%)	133 (19.9%)	145 (21.7%)	87 (13.0%)

* Multiple responses

**Test of the study hypotheses:** to guide this study, seven hypotheses were formulated and were tested to determine if there was a statistically significant association between school absenteeism by the girls during the menstrual period and perceived associated factors.

### Hypothesis 1

There is no statistically significant difference between menstruating rural and urban adolescent in-schoolgirls in Cross River State who miss school due to the fear of being ridiculed by other students. The result of the test of this hypothesis, as shown in Table 3, shows that there is a statistically significant association of school absenteeism of our respondents with the fear of being made fun of or ridiculed by fellow students. X2=15.398 (1), p ≤ 0.001; and that location was a determining factor. While 91.3% urban and 80.5% rural-based adolescent in-schoolgirls respectively agreed that teasing was responsible for school absenteeism during their menstrual period, 8.7% urban and 19.5% rural-based girls disagreed. Also, from Table 2, it was shown that the effect of teasing had greater effect on urban-based students (60.1%), compared to their rural-based counterparts (27.5%).

**Table 3:** chi-square analysis of factors associated with missing school during menstruation among adolescents in-school girls in CRS, Nigeria

Reasons for missing school	Pearson Chi-square value	df	Asymp. sig. (2-sided)
I am afraid of others making fun of me by Location of School	15.398	1	0.001*
I am afraid of staining my school uniform by location of school	3.277	1	0.070
I do not have sanitary towels by location of school	7.462	1	0.006*
I have pains by location of school	3.428	1	0.064
I do not have sanitary towels by location and mothers´ socioeconomic status	8.810	2	0.012*

Statistically significant tests of associations (p<0.05) Critical X2 (3.841)

### Hypothesis 2

There is no statistically significant difference between rural and urban-based adolescent in-schoolgirls in Cross River State missing school due to the fear of staining their school uniform. The result of the test of this hypothesis indicated that the fear of staining their school uniform during their menstrual period was also not statistically significantly associated with their missing school X^2^= 3.277 (1), p = 0.070 (Table 3).

### Hypothesis 3

There is no statistically significant difference between rural and urban-based in-school adolescent girls in Cross River State who miss school due to lack of sanitary towels. The result of the test of the hypothesis showed that the lack of sanitary towels was significantly associated with school absenteeism; X^2^= 7.462 (1), p = 0.006 (Table 3). Moreover, the respondents´ location was found to be associated with the lack of sanitary towels. A greater proportion of urban girls, 356 (58.2%) missed school as a result of the lack of sanitary towels compared to 158 (25.8%) rural-based respondents (Table 2).

### Hypothesis 4

There is no statistically significant difference between rural and urban-based adolescent in-schoolgirls in Cross River State missing school due to menstrual pain. The result of the Chi-squared test of association showed that there was no statistically significant difference in school absenteeism between the urban and rural-based respondents as a result of menstrual pain. X^2^=3.428 (1), p = 0.064 (Table 3).

### Hypothesis 5

There is no statistically significant association between lack of sanitary towels among adolescent in-schoolgirls in Cross River State who miss school during their period and the socio-economic status of their mothers. The Chi-Square test of association for this hypothesis showed that there is a statically significant association between the lack of sanitary towels among the adolescent girls and the socio-economic status of their mothers: X^2^= 8.810 (2), p = 0.012 (Table 3).

**Figure 2:**
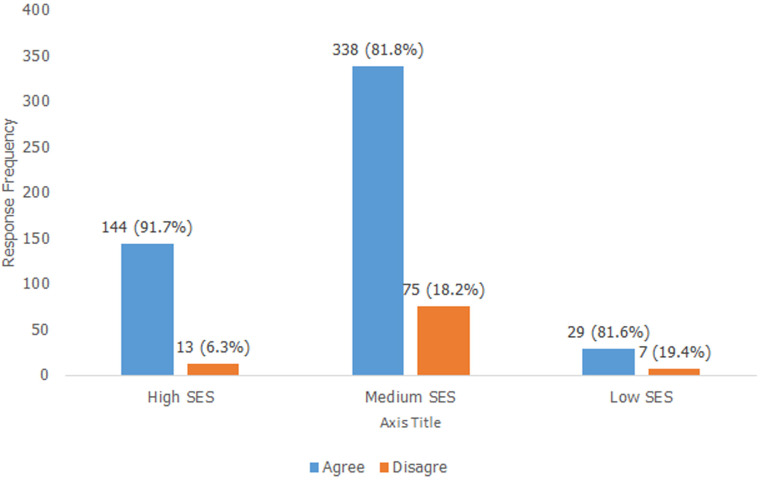
missing school during menstrual period due to lack of sanitary pad by mothers´ SES

Moreover, Figure 2 shows that the lack of sanitary towels expressed by the adolescent girls cut across the three socio-economic classes with some rural-urban disparities in access to sanitary pads among the high, medium and low income groups. The proportion of respondents in the urban-based schools who missed school due to lack of sanitary pads was higher, (27.9%) for the girls in urban schools whose mothers were classified as high socioeconomic status (SES) compared to their counterparts in the rural area (15.2%). On the contrary, the proportion of girls in the rural areas whose mothers belonged to the medium SES category, who missed school for having no access to sanitary pads (57.9%), compared with their counterparts in the urban setting (54.8%) (Figure 2).

## Discussion

Menstruation is a normal physiologic process in women characterized by a monthly cyclical blood flow resulting from the shedding of the lining of the womb. However, in some low- and middle-income countries, the subject of menstruation is often shrouded in the culture of silence that forecloses any public or open discussion of the issue [12, 13]. This negative societal disposition towards menstruation, imposes some limits on the ability of girls and women to fully and equally partake in societal affairs, resulting in limited access to appropriate information on menstruation and its management. Above all, it undermines their overall self-esteem and social status [14]. A 2015 study by the United Nations Children´s Fund (UNICEF) conducted in three Nigeria States, namely: Katsina, Osun and Anambra, brought to the fore the fact that schoolgirls in Nigeria face great discomfort during their menstrual period in addition they experience anxiety, abdominal cramps and pain, nausea, vomiting, dizziness, and a loss of appetite; resulting in their missing classes. The study also revealed that the availability of Water, Sanitation and Hygiene (WASH) facilities in the schools were grossly inadequate for menstrual management; and that only about 41.7% of the toilets in the school had functional locks, while only about 25% of the schools had stands with hand-washing basins and soaps [15]. In a mixed-method study on menstrual health and school absenteeism among 352 Ugandan adolescent girls, with median age of 16 years, Miiro *et al*. [16], reported that 64 (18.7%) of the girls acknowledged that during their most recent period, they stained their clothes and consequently missed at least 1 day of school. In a sub-study that was conducted among 40 girls, in which the girls recorded their menstrual experiences in a diary, missing school by the girls was reported on 28% of the menstrual period-days, compared with 7% of period-free days (the adjusted odds ratio=5.99, 95%CI: 4.4, 8.2; p < 0.001); indicated that among these girls, school absenteeism was strongly associated with menstruation. Evidences from other studies [17-21] have equally shown that adolescent schoolgirls face lots of challenges during their monthly menstrual periods often resulting in school absenteeism. The current study was therefore conducted to identify factors that cause menstrual period-related school absenteeism among urban and rural adolescent secondary school girls, age between 10 to 18 years in Cross River State, Nigeria.

### Missing school as a result of menstrual pains and feelings of discomfort

Menstruation is a painful experience and for girls who have this experience for the first time, it can be an extremely frightening one. Mohammed *et al*. [22], in a school-based mixed-model cross-sectional study conducted in five Junior High Schools in a rural community in Ghana, involving 250 adolescent schoolgirls, to assess their understanding of menstrual hygiene management (MHM), and how menstruation influences school absenteeism; the researchers found that of the 101 respondents who experienced menstrual-related absenteeism, menstrual pain was a major factor. (n = 83, 82.2%). The result of a study by Sivakami *et al*. [23] conducted to compare menstrual hygiene practices and challenges between publicly owned schools and UNICEF WASH-support Model schools in three Indian states (Tamil Nadu, Maharashtra and Chhattisgarh), among menstruating schoolgirls, above 12 years (N=492 for model schools, and (N=2072, for Public schools), showed that in both model vs. public school respectively, menstruation with associated pain was (31% vs 38%, P = 0.004) while menstruation associated with fear of stain or smell, was (11% vs 16%, P=0.002). The result indicated that the Model WASH schools had fewer reports of painful menses and shyness/self-stigmatization associated with menstruation compared with the public schools. From our study, about 67% of our respondents (Table 1) indicated that menstrual pains and the feeling of discomfort played some role in their absenting themselves from school during their monthly cycle. Our study indicated that urban or rural location was not a determinant of menstrual pain, as it was common to both urban and rural-based girls.

### The role of socio-cultural stigmatization and misconceptions about menstruation

Cultural and social norms about menstruation affect school attendance by girls in Nigeria [24]. Misconceptions about menstruation based on cultural and religious norms and practices that bar menstruating girls/women from engaging in every-day routine activities, such as: cooking, education and working are still prevalent in Nigeria [25, 26]. These entrenched norms and the fear of ridicule make the girls apprehensive when they have their period, and may account for the self-imposed school absenteeism by the adolescent girls during their periods. Mohammed *et al*. [22], in their study involving 101 subjects, found that 71 (70.3%) of the students absented themselves from school during their period for fear of being ridiculed by their male colleagues for staining their uniforms. Tegegne *et al*. [27] in a mixed-method research conducted in Northeast Ethiopia that included 595 randomly selected adolescent schoolgirls, with mean age at menarche of 13.98 (± 1.17) years, reported that more than 50% of the girls acknowledged missing school during their menstrual period for fear of being ridiculed or embarrassed for staining their school uniform as a result of the non-usage of sanitary napkins. In our study, 522 (51.9%) and 552 (54.9%) girls of the total, 1006 respondents agreed respectively that they stayed away from school during their period because they were afraid of staining their school uniforms and being ridiculed by fellow students. The girls may have felt compelled to stay away from school because of the cultural stigma of uncleanness associated with menstruation [28, 29].

### Lack of sanitary products

Tegene *et al*. [27] in their study in Northeast Ethiopia, showed that the risk of an adolescent schoolgirl who did not use a sanitary material missing school during her menses was about five times the risk of her counterpart who used a sanitary material; [AOR-95% C.I: 5.37 (3.02 - 9.55)]. Our study found a statistically significant association between the lack of sanitary towels and the following factors respectively: urban vs. rural location of the respondents (X2 =7.462 (1), p = 0.006), the mothers´ socio-economic status, (X2 = 8.810 (2), p =0.012. Moreover, majority 358 (58.2%) of the urban-based adolescent girls expressed the lack sanitary towels as a hindrance compared to 158 (25.8%) of their rural-based counterparts, who most probably may use other forms of absorbent materials like handkerchiefs or pieces of cloth, which are often washed and reused. According to the World Poverty Clock, an estimated 87 million Nigerians are said to live in extreme poverty; and it is projected that by 2030, a total population of 120 million Nigerian will fall below $1.09 per day, if the current trend continued [30]. In Nigeria, menstrual products are taxed, which increases the price and puts extra financial pressure on adolescent girls from poor families and communities. The high cost of sanitary towels in Nigeria, which cost an average of N471.90 ($1.30) per pack, places a major constraint on the girls who cannot afford the towels and therefore cannot effectively manage their menstrual period while at school [31].

### Socio-economic determinants of lack of sanitary products

Ideally, it is expected that mothers would be more concerned about the menstrual hygiene management of their daughters; and would strive to provide the basic requirements for the MHM of their wards. Findings from the study, as shown in Figure 2, indicated that there was a lack of access to sanitary towels which cut across all the three social class categories of the mothers assessed; namely: high social class for mothers who were civil servants, medium class for self-employed businesswomen, while the low social class category was applied to peasant women and those with no formal employment. The data showed that majority 144 of the 157 respondents i.e. (91.7%) from both rural and rural settings, whose mothers belonged to the high social class, lacked sanitary towels, this was also applicable to 81.8% (i.e. 338 out of 413) of the students whose mothers were categorized as belonging to the medium social class. Finally, 29 of the 36 respondents (i.e. 80.6%) of the girls who lacked access to sanitary towels also belonged to the low social class category. The overall result therefore showed that the lack of sanitary towels by the girls for their MHM, cut across the three social classes. This may be a reflection of the income levels of the mothers of these in-school adolescent girls. Parental income is therefore a critical determinant factor for both urban and rural based adolescent students in having access to sanitary wares and a key factor in school absenteeism.

### Institutional and systemic factors

Globally, an estimated 500 million women and girls do not have access to adequate MHM facilities. The poor access to WASH facilities, especially in public places, such as schools, markets, offices or health centers, constitutes a major hindrance to the attainment of an equitable sexual health for women and girls. Evidences from studies have shown that the inability of girls to effectively manage their menstrual period within the schools setting, promotes school absenteeism. This, in turn, affects their development and aspiration and invariably imposes severe economic costs on their lives and by extension the country [22, 32-34]. An association has also been found to exist between absenteeism of student and unfavorable school setting conditions [33], such as lack of WASH facilities or privacy.

Moreover, the findings of a World Bank study on Nigeria showed that 25 percent of women lacked adequate privacy for managing their menstrual periods and also for defecation [35]. This corroborates our findings in the schools where majority (75%) of the girls indicated that they missed school as a result of the absence of WASH facilities for menstrual hygiene management. Similar findings were reported for Bangladesh and Panama [36]. The issue of missing school by adolescent schoolgirls goes beyond just stigmatization and the misconception of menses as an ailment. The pressing concern for many families in developing countries is about their livelihoods; how to keep body and soul together, and for most families in Nigeria, who are living below the poverty line [37], things like sanitary wares are considered to be either non-essentials or luxuries; as such girls from such families may miss school during their period because their parents cannot afford to buy them sanitary wares.

In sub-Saharan African the market for sanitary towels is projected to reach $779 million (282 billion Naira) by year 2022, thereby creating the euphemism that has become known as “period poverty” for those who are a due to financial inequality and other socio-economic constraints cannot afford to buy sanitary wares for their menstrual hygiene management during their period [31]. In addition to contending with myths and taboos about menstruation [38], educational institutions in Nigeria should also be equipped with the capacity to deal with the pain and physical discomfort associated with menstruation. Schools should be provided facilities to deal with symptoms associated with menstruation such as anxiety, dizziness, abdominal pain, cramps, nausea, vomiting, and loss of appetite [39].

**Limitations of the study:** the study has some limitations that should be taken into account when making inferences about the findings. Firstly, data on household income were not collected. Data on parents´ occupation were used to determine the socio-economic status of the parents. Secondly, as a cross-sectional study, the likelihood of recall bias cannot be ruled out.

**Funding source:** the study was funded to the Tertiary Education Trust Fund (TETFUND) Institutional Based Research (IBR) Projects, Abuja, Nigeria, Ref: UC/AP.185.

## Conclusion

The study has brought to the fore various reasons why adolescent in-schoolgirls in Cross River State miss school during their menstrual periods. Among the key factors identified include phobias about staining their school uniform, ridicule by male colleagues, enduring the discomfort of the physiological effects of menses such as menstrual pains and the sickening feelings of fatigue; as well as coping with institutional or systemic problems such as the lack of sanitary towels and WASH facilities where they can wash and change or even dispose their soaked sanitary wares in a dignified, safe and healthy manner. School absenteeism can impact negatively on the girl-child´s overall academic performance, intellectual development and aspirations in life. Consequently, there is need for adequate sexuality education support and interventions to prevent stigmatization, eliminate shame and promote self-esteem among girls moreover, endemic poverty in Nigeria should also be addressed, while schools should have adequate WASH facilities to eliminate the stress associated with menstrual hygiene management by adolescent girls while at school.

### What is known about this topic

- School absenteeism due to menstrual pain, ridicule and lack of menstrual pad has been well documented by various studies in Africa;
- However rural-urban differentials in school absenteeism has not been well documented.

### What this study adds

- This study showed that within the Nigerian context socio-economic status and parental income should be taken into consideration in addressing MHM issues of adolescent in-schoolgirls in both rural and urban settings with regards to school absenteeism during their menstrual periods.
